# Response of Soil Detachment Rate to Sediment Load and Model Examination: A Key Process Simulation of Rill Erosion on Steep Loessial Hillslopes

**DOI:** 10.3390/ijerph20042839

**Published:** 2023-02-06

**Authors:** Nan Shen, Zhanli Wang, Fengbao Zhang, Chunhong Zhou

**Affiliations:** 1 State Key Laboratory of Soil Erosion and Dryland Farming on the Loess Plateau, Institute of Soil and Water Conservation, Northwest A&F University, Xianyang 712100, China; 2 State Key Laboratory of Soil Erosion and Dryland Farming on the Loess Plateau, Institute of Soil and Water Conservation, Chinese Academy of Sciences and Ministry of Water Resources, Xianyang 712100, China

**Keywords:** soil erosion model, soil detachment, sediment load, WEPP model, EUROSEM model, rill flume experiment

## Abstract

The rate of soil detachment by water flow indicates soil erosion intensity directly. The exact relation between soil detachment rate and actual sediment load in water flow, however, is still unclear, and the existing relationships have not been adequately tested. The aims of the present study were to investigate the response of soil detachment rate to sediment load using rill flume data with loessial soil and to quantitatively examine the soil detachment equations in the WEPP and EUROSEM soil erosion models. Six slopes were combined with seven flow discharges to measure detachment rates under seven sediment loads using a rill flume with a soil-feeding hopper. Significant differences were found among the soil detachment rate by different sediment loads in low sediment load levels, but an insensitive response of soil detachment rate to sediment load was found under high levels of sediment load. The soil detachment rate was proved to be negatively linearly correlated with sediment load. The rill detachment equation in the WEPP model predicted the soil detachment rate by rill flow very well under our experiment condition. The soil detachment equation in the EUROSEM model underestimated the detachment rates under controlled conditions, but removing the setting velocity from the equation greatly improved prediction. Further experiments that could reflect the dynamic convective detachment and deposition process need to be conducted to compare with the present examination results and to further understand rill erosion processes.

## 1. Introduction

Soil erosion is a serious environmental problem worldwide, and results in the area of cultivated land decreasing, the soil quality declining, sediment deposition on riverbeds, and serious flooding disasters [1,2,3,4,5,6,7]. All the environmentally harmful consequences of soil erosion greatly threaten the sustainable development of human beings. Physically based prediction models of soil erosion are of great significance for erosion hazards prevention, the development of which requires a good understanding of the soil erosion process. Rill erosion is an important soil erosion type on hillslopes [8,9,10], and soil detachment of soil particles from the soil body by rill flow and sediment transport by rill flow are the key processes of rill erosion [11]. The rate of detachment directly indicates soil erosion intensity [12], and the soil detachment process provides sediment sources for transport processes. Along with the increase in sediment load in rill flow, the sediment transport process, in turn, may have feedback effects on the soil detachment process. However, the influence and relation of the actual sediment load runoff transported on the soil detachment rate is still ambiguous, and the existing detachment model equations have not been adequately tested. The relationship between soil detachment rate and sediment load in rills thus need to be investigated for a good understanding of the rill erosion process and for developing physically based prediction models of erosion. Further, the existing equations in soil erosion models need to be examined to evaluate their applicability.

Meyer and Wischmeier [13] divided the soil erosion process into soil detachment and sediment transport. Field experiments of Huang et al. [14] concluded that the soil detachment rate was not affected by sediment load; treating the two processes individually was necessary to understand of the erosion mechanism. In contrast, the spatial distribution data of the soil detachment rate and sediment concentrations showed that the detachment rate decreases linearly with the sediment load [12]. Moreover, two soil detachment equations, in which the sediment load was introduced as a factor, were widely used to predict the soil detachment rate: soil detachment equation in WEPP (Water Erosion Predict Project) [15] and soil detachment equation in EUROSEM (European Soil Erosion Model) [16].

The soil detachment equation proposed by Foster and Meyer in 1972 stated that the detachment rate decreased with an increase in sediment load. This function, also known as the sediment feedback relationship, was introduced in the WEPP model for soil detachment prediction:

(1) $$D_{r}=D_{c}(1-\frac{q_{s}}{T_{c}})$$

where *D_r_* is soil detachment rate in rills (kg m^−2^ s^−1^), *Dc* is the detachment capacity by rill flow (kg m^−2^ s^−1^), *q_s_* is the actual sediment load in rill flow (kg m^−1^ s^−1^), and *Tc* is the sediment transport capacity (kg m^−1^ s^−1^).

EUROSEM estimates the detachment rate based on a famous erosion–deposition theory [17]. Assuming that erosion and deposition are two continuous counteracting processes, the transport capacity of the runoff represents the sediment concentration at which the rate of erosion by the flow and the accompanying rate of deposition are equal. The net detachment rate is zero under this balanced condition, and the erosion rate equals the deposition rate (*wv_s_T_c_*). The detachment rate was thus proportional to the transport capacity deficit:

(2) $$D_{r}=\beta wv_{s}(T_{c}-q_{s})\;$$

where *D_r_* is the net detachment rate of soil particles by flow (m^3^ s^−1^ m^−1^), β is a flow detachment efficiency coefficient correlated with soil cohesion, *w* is flow width (m), *v_s_* is setting velocity of soil particles (m/s), *T_c_* is the transport capacity (m^3^ m^−3^), and qs is the actual sediment load (m^3^ m^−3^).

Equation (1) shows that the detachment rate increases negatively with the sediment load. A linear equation between two extreme cases of clear water (*q_s_/T_c_* = 0) and the maximum sediment load (*q_s_/T_c_* = 1) was assumed [18]. The rate of detachment is maximal and is equal to the detachment capacity when *q_s_* = 0, which means the entire energy of rill flow is used to detach soil in clear water conditions. The rate of detachment is zero when *q_s_* = *T_c_*, which means total energy of rill flow is used for sediment transport in maximum sediment load conditions. The rate of detachment is greater than 0 and smaller than Dc when 0 < *q_s_* < *T_c_*, when part of the flow energy is used to sediment transport, and the remaining energy is used to detach soil from the bed.

The rare earth element experiment [19] and flume experiment [20] confirmed that the phenomenon of sediment feedback existed and the detachment equation in WEPP model (Equation (1)) was accurate. However, there are still some outstanding problems. On the one hand, whether the detachment equation in WEPP could represent the exact relationship between detachment and transport was uncertain [21], because parameter estimation was difficult at low rates of sediment inflow [22]. On the other hand, an evaluation of flow detachment on smooth and natural beds found that the soil detachment process and sediment transport process were driven by different hydrodynamic parameters [23], so whether the two process could be connected by a simple first-order coupling relation was uncertain.

A framework for the interaction of erosion and deposition assumed that detachment by flow included detachment from the original soil body and re-detachment of soil particles from the deposited layer [24,25,26], whereas the re-detachment from a deposited layer was not used in the EUROSEM models. Knapenet et al. [27] proposed that the detachment relationship in EUROSEM had the advantage of simplicity to predict detachment rates. However, simply measuring soil cohesion only is not accurate for describing the temporal and spatial variation of erosion resistance.

In summary, the relationship between detachment rate and sediment load still needs to be investigated, and the two detachment equations in the WEPP and EUROSEM models require experimental examination. Loess is easily eroded [28,29], and the flow hydraulic on steep slopes differ from those on gentle slopes [19,30,31,32]. Steep slopes represent more complex hydraulic conditions and serious erosion, but the relationship between detachment rate and sediment load have rarely been studied with rill-flume data on steep loessial hillslopes, and the existing model relationships have not been adequately experimentally examined to evaluate their applicability for these typical conditions of erosion on the Loess Plateau of China. This study aims to investigate the response of soil detachment rate to sediment load using rill-flume data on a simulated steep loessial hillslope, and to quantitatively examine the soil detachment equations of the WEPP and EUROSEM erosion models.

## 2. Materials and Methods

### 2.1. Soil, Equipment, and Experiment Design

The soil used in this study was loessial soil. The particle size distribution of the test soil was shown in Table 1. The test soil belongs to sandy loam base on the soil particle size classification of United States.

**Figure 1:**
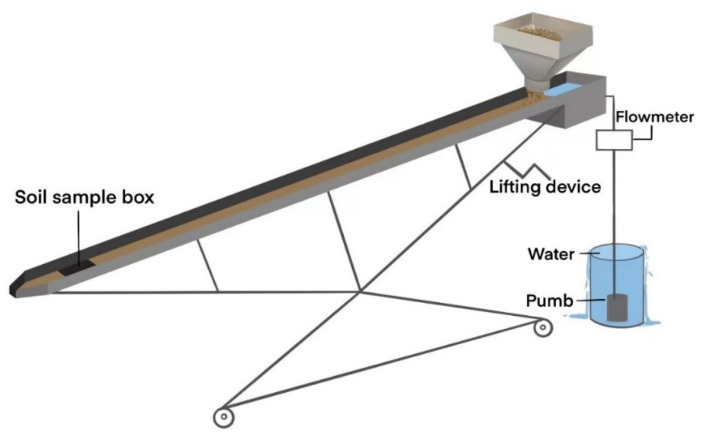
Experimental equipment.

Rill flume experiment with a soil feeding hopper was used in this study. Figure 1 shows the experimental equipment. The rill flume is 4m in length, 0.1 m in height, and 0.1 m in width; the section of the rill flume is rectangular rill geometry. In the upper tail end of the rill flume, a soil feeding hopper was installed. The soil feeding hopper has a built-in rotor, the rotation of which was controlled by electric motor so as to adjust the soil-feeding rate. In the downstream of the rill flume, a square cavity was dug and a soil sample box 0.1 m in length and width, 0.05 m in height could be put in it suitably. The soil in the soil box was detached by rill flow to obtain the soil detachment rate. A lid was used to control the beginning and end of soil detachment by covering it in the soil box or not. The flume bed was brush with a layer of glue and then had a layer of test soil spread upon it to simulate the natural slope surface. A flow meter was used to control the discharge of rill flow.

To build the equipment, 7 units of flow discharges, 6 slopes, and 7 sediment loads were completed combined, and each treatment was repeated once. The detailed experimental conditions are shown in Table 2.

### 2.2. Measurement of Sediment Transport Capacity (T_c_)

The value of *T_c_* by rill flow was the basis to adjust the soil feeding rate of the hopper, and was also the necessary data for model testing. Firstly, the soil box filled with loose experiment soil was saturated with water for 12 h, then placed into the square cavity and covered with a lid; the rill flume slope and flow discharge were also correctly set to a designed combination. Secondly, the soil feeding rate of the hopper was gradually regulated, following increasing sequences until the feeding soil could not be completely transported and a very small amount of deposition occurred, at which point we recorded the soil feeding rate. At the same moment, the lid covering the soil box was removed. The soil box was a double insurance to make sure the rill flow was saturated with sediment, which could decrease the error caused by human observation. The soil in the soil box might otherwise be detached if there was a little sediment deficit or might be maintained if the *Tc* of rill flow has been reached. Finally, five samples of sediment laden rill flow were collected for each test, and the duration of each sampling was timed. The collected samples were allowed to stand for 12 h and the clear supernatant was discarded. The wet soil was oven-dried and the dry soil was weighed. The *T_c_* (kg m^−1^ s^−1^) was calculated as the dry soil weight of sample divided by the sampling time (s) and flume width (m). Overall, 42 *T_c_* were obtained in this measurement section.

### 2.3. Measurement of Soil Detachment Rate under Various Sediment Loads

Firstly, the soil box was filled with test soil in two soil layers with a volume weight of 1.2 g cm^−3^ and soil water content of 14%—which was designed to simulate nature soil conditions of rill erosion—and saturated with water for 12 h. The bulk density, soil water content, and the volume of soil sample box were fixed, so the amount of soil in the soil sample box was the same for each test. The ready soil sample was left in the square cavity. Secondly, the slope of the rill flume, flow discharge, and sediment load were correctly adjusted. The sediment load was designed as a different percent of *T_c_*, and the soil feeding rate was adjusted based on the sediment transport capacity measured in the Section 2.2 to produce a different sediment load. Finally, the lid on the soil box was moved away, then the soil was detached. The detachment process was stopped by using the lid to cover the soil box once again when the detachment depth reach was almost 2cm, and the duration of detachment was recorded. The detachment rate (kg m^−2^ s^−1^) was calculated as the dry soil weight that has been detached (kg) divided by the duration of detachment (s) and the projected area of soil sample (m^2^). A series of 294 soil detachment rates were obtained.

### 2.4. Parameter Calculation

Soil cohesion was measured using a shear apparatus (14.10 Tester) under saturated conditions, and the flow detachment efficiency coefficient, *β*, was calculated based on the soil cohesion (*J*, kPa) under saturated conditions. For *J* < 1, *β* is assumed to be 0.335. For *J* > 1, *β* is reduced exponentially as follows [33,34]:

(3) $$\beta =0.79e^{-0.85J}\;$$

Settling velocity, *v_s_*, was obtained by the following method proposed by Cheng [35]:

(4) $$\frac{v_{s}d}{\nu }={\left(\sqrt{25+1.2d_{*}{}^{2}}-5\right)}^{1.5}$$

where *d* is the particle diameter (m), *v* is the kinematic viscosity (m^2^ s^−1^), the water temperature was measured to determine it for each trial, and $d_{*}$ is a dimensionless particle parameter given by the following:

(5) $$d_{*}={\left(\frac{(\rho_{s}-\rho )g}{\rho \nu^{2}}\right)}^{1/3}d\;$$

where *ρ_s_* is particle density, *ρ* is fluid density, and *g* is acceleration due to gravity.

The statistical parameters were calculated in SPSS 18.0 and excel 2016. The figures were drawn in origin 2021b.

## 3. Results

### 3.1. Response of Soil Detachment Rate to Sediment Load

**Figure 2:**
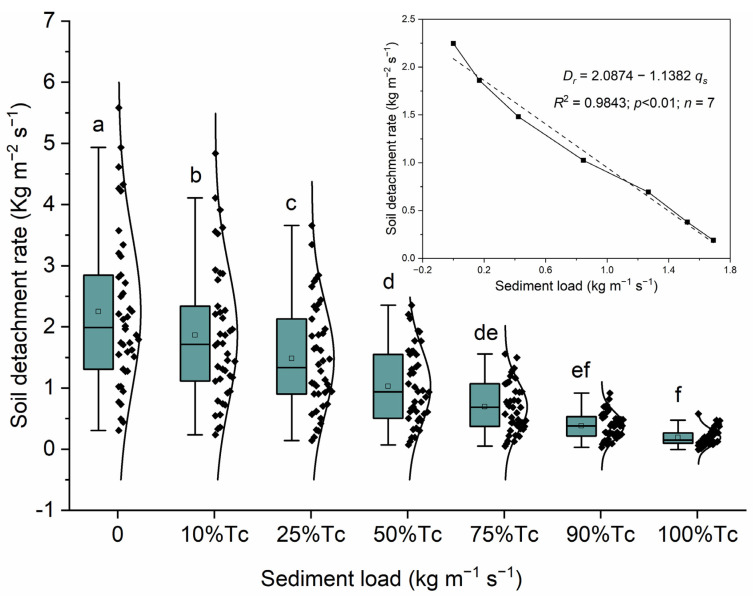
Soil detachment rate by sediment-laden rill flow under different levels of sediment load.

The soil detachment rate (*SDR*) by sediment-laden rill flow under each level of sediment load including 0, 10% *T_c_*, 25% *T_c_*, 50% *T_c_*, 75% *T_c_*, 90% *T_c_*, and 100% *T_c_* were statistically compared by a one-way analysis of variance, as shown in Figure 2. Significant differences were found among the soil detachment rates under low levels of sediment load (0~50% *T_c_*). For the soil detachment rate under high sediment load, there were no significance differences between the soil detachment rate under 50% *T_c_* and 75% *T_c_*, as well as that under 75% *T_c_* and 90% *T_c_*, and 90% *T_c_* and 100% *T_c_*. However, significant differences were found between the soil detachment rate under 50% *T_c_* and 90% *T_c_*, and under 75% *T_c_* and 100% *T_c_*. Therefore, *SDR* responded sensibly to sediment load when the portion of rill flow filled by sediment had a low value, that is to say, a small increase in sediment load of rill flow could produce an obvious decrease in soil detachment rate when rill flow had a big sediment deficit. On the contrary, when the actual sediment load was close to *T_c_*, the response of *SDR* to sediment load was not so sensitive; a significant decrease in soil detachment rate occurred only when a big increase in sediment load was given.

Additionally, after the data were analyzed by regression, soil detachment rate was proved to be negative linear correlated with sediment load (*D_r_* = *a* − *bq_s_*) (Figure 2). *R*^2^ for the linear equations was 0.9843 (*p* < 0.01). This linear equation also demonstrated two extreme cases. First, the *SDR* under clear rill flow (*q_s_* = 0) was the largest, indicating that clear water detached the most soil, terming the soil detachment capacity. Second, the *SDR* decreased with the increase in sediment load until it approached zero at the maximum sediment load, i.e., *T_c_*.

### 3.2. Examination of WEPP Rill Detachment Equation

The soil detachment equations of the WEPP model and EUROSEM model link the soil detachment rate and sediment load with negative relationships, as shown in Equations (1) and (2). We thus examine these models to verify the feasibility of the models when compared to loess hillslopes. The soil detachment equation of the WEPP model was examined first. The datasets of our study were divided into gentle slopes (6°, 9°, 12°) and steep slopes (15°, 18°, 21°), and thus the performances of the WEPP model under gentle slopes, steep slopes, and all slopes (6°, 9°, 12°, 15°, 18°, 21°) were examined. The measured values of each independent factor in Equation (1) were used to calculate the predicted soil detachment rate by rill flow for comparison with the directly measured value of *SDR* by sediment-laden rill flow.

**Figure 3:**
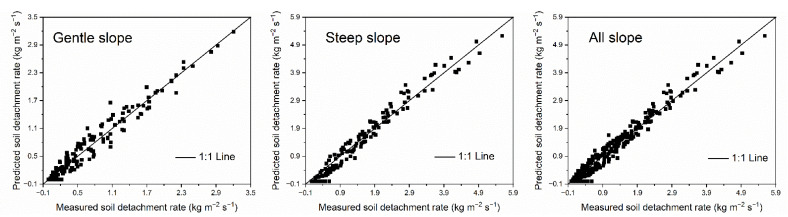
Measured vs. predicted detachment rates using the soil detachment equation in WEPP for 294 trials.

The performance of the WEPP soil detachment equation under different slope conditions was shown in Figure 3. The predicted soil detachment rate (*SDR*) using the equation in the WEPP model, on the whole, matched the measured *SDR* well for gentle slopes, steep slopes, and all slopes. Compared with the data points of gentle slopes, the data points of steep slopes focused more closely around the 1:1 line, which indicated that the performance of the WEPP soil detachment equation on steep slopes was better than that on gentle slopes. The statistics between measured and predicted data using the detachment equation in WEPP were shown in Table 3. The *RE*, *MARE*, *R*^2^ and *NSE* also indicated that the prediction accuracy of WEPP soil detachment equation for steep slopes was better than that for gentle slopes, as well as the prediction accuracy for all slopes. For gentle slopes, *RE* ranged from −119.7683 to 100, *MARE* was 35.57, *R*^2^ was 0.9516, and *NSE* was 0.9484. By contrast, these values were −47.1623 to 100, 29.24, 0.9681, and 0.9572, respectively, on steep slopes. In summary, the detachment equation in WEPP accurately estimated the *SDR* on loess hillslopes, and the applicability of the model to steep loess hillslopes was better than that on gentle loess hillslopes. The response relation of *SDR* to sediment load could be well described by the soil detachment equation in the WEPP model.

### 3.3. Examination of the EUROSEM Soil Detachment Equation

**Figure 4:**
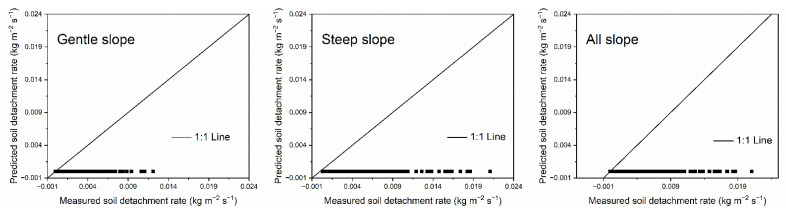
Measured vs. predicted detachment rates using the soil detachment equation in EUROSEM for 294 trials.

The units for soil detachment rate and sediment load differ in the EUROSEM and WEPP model, so we transformed the data to fit the units of EUROSEM model to evaluate the performances of the EUROSEM soil detachment relationship under gentle slopes, steep slopes, and all slopes. The measured value of flow detachment efficiency coefficient (*β*), settling velocity (*v_s_*), width of the flow (*w*), actual sediment load (*q_s_*), and sediment transport capacity (*T_c_*) were introduced into Equation (2) to calculate the predicted *SDR* for comparison with the measured data. Results showed that the EUROSEM soil detachment relationship produced large errors between the measured and predicted detachment rates under every slope condition (Figure 4, Table 4). The values of *NSE* were in the range of −1.1312~−1.5918. The points of soil detachment rate distributed far away from the 1:1 line; these data points all fall in the area below the 1:1 line, which indicated that *SDR* was badly underestimated by the EUROSEM soil detachment equation. The EUROSEM detachment equation based on erosion–deposition theory could not accurately predict the detachment rates on the simulated loess hillslopes at the various sediment loads in this experiment.

## 4. Discussion

### 4.1. Negative Feedback Effects of Sediment Load on SDR

The detachment process provides a sediment source for the transport process, and along with the rising sediment load in rill flow, the sediment transport process, in turn, has negative feedback effects on the soil detachment process. The results of Zhang et al. [12], Zhou et al. [36], and Zhang et al. [19] also reported the negative relationship between the SDR and sediment load. The negative correlation with the sediment load was primarily due to the change in flow energy distribution [19,20] and the change in flow turbulence during rill erosion [37]. The energy contained in the rill flow is mainly used to detach soil from the soil mass and transport soil particles. More flow energy expended on detachment produces a higher detachment rate at a constant critical shear stress and soil erodibility. A rising sediment load, however, causes a bigger energy consumption for sediment transport and less for soil detachment [36]. The SDR thus decreased with sediment load. In addition, flow turbulence has a big positive contribution to the soil detachment. However, the value of the Reynolds number and Froude number may decrease with an increase in sediment load [38], thus increasing sediment load in rill flow may weaken the turbulence of rill flow [39]. Consequently, sediment transport performs negative feedback effects on the soil detachment process by rill flow. Moreover, there still a factor named sediment shield that explains that sediment in rill flow may cover the soil bed during erosion, so shielding the soil from runoff may thus reduce the soil detachment rate [20,40].

### 4.2. Investigation of the Underestimation Prediction by EUROSEM

The reason for the unreasonable prediction by EUROSEM was investigated. Negative linear regression equations describing the response relation of *SDR* to sediment load were established (Table 5). The consistencies of the regressive intercept a in the regression equations of Table 5 and the combined parameter *βωv_s_T_c_*, as well as the absolute value of the regressive slope b and the combined parameter *βωv_s,_* were compared. Statistical comparisons indicated that the combined parameters were three orders of magnitude lower than the regressive values of intercept *a* and slope *b*. Further investigation of the original experimental data also illustrated that the rate of soil detachment was underestimated by three orders of magnitude using the EUROSEM detachment model. Coincidentally, the average settling velocity, *v_s_*, was near to 0.001. Therefore, we deduced that the parameter average settling velocity led to the underestimation of the EUROSEM soil detachment equation.

**Figure 5:**
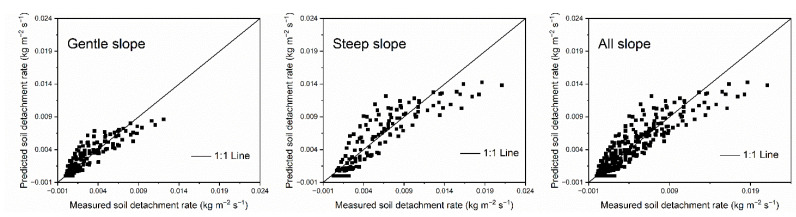
Measured vs. predicted detachment rates using the revised EUROSEM soil detachment equation without *v_s_* for 294 trials.

Detachment and deposition are two continuous counteracting processes in the erosion-deposition theory of EUROSEM [16], and settling velocity in the detachment equation is a key parameter representing deposition. The *SDR* was thus recalculated using a revised Equation (2) without *v_s_* and then compared with the measured data. Results showed that removing *v_s_* from the equation greatly improved the agreement between the predicted and measured detachment rate (Figure 5, Table 6); the values of *NSE* rose to the range of 0.7602~0.7984 for different slope conditions. The inaccurate predictions of Equation (2) and improved predictions of the revised Equation (2) without *v_s_* further prove that the underestimation of EUROSEM soil detachment equation is thus due to the parameter of settling velocity. The role of settling velocity in the EUROSEM detachment equation must be taken into consideration to avoid excessive underestimation.

Discussing the underestimation from the perspective of experimental design, the deposition was weak under our experimental conditions. Because a non-erosive flume bed was used in this study, the erosion area of the flume bed designed in this study was a small area, and the sediment in the rills was not eroded soil but soil fed from the hopper, so the simulated detachment rates at different sediment loads were quantitatively accurate, but the dynamic convective detachment and deposition may not have been completely expressed. The incompatibility of the weak deposition under experimental conditions with the erosion–deposition theory of EUROSEM accounted for the large underestimation. However, the deposition rate in this study was not being measured. On the one hand, this experiment highlights that it is important to investigate the fundamental cause of unreasonable prediction for a fairness examination. On the other hand, further study must be undertaken to explore methods for measuring deposition rates to test EUROSEM accurately, and to determine if models based on different theories need different experimental designs to test. Moreover, the rill erosion process and the development of rill in the field may be different with indoor simulation experiment data [41,42]; quantitative studies on the effect and mechanism of upslope sediment load on soil detachment in rills must be conducted in the field in future study.

## 5. Conclusions

The response of the soil detachment rate to sediment load and model examination was studied by flume experiment at several slopes, flow discharges, and sediment loads using loessial soil. Significant differences were found among the soil detachment rates under low levels of sediment load, but an insensitive response of soil detachment rate to sediment load was found under high levels of sediment load. The soil detachment rate was proved to be negatively linearly correlated with sediment load. The rill detachment equation applied in the WEPP model correctly described the detachment and transport processes during erosion and had a good applicability to steep loessial hillslopes. The soil detachment equation in the EUROSEM model underestimated the detachment rates under controlled conditions, but removing the setting velocity from the equation greatly improved prediction, which indicated that the weak deposition under experimental conditions may lead to underestimation. Experiments that could reflect the dynamic convective detachment and deposition process must be conducted to compare with the present examination results and to further understand rill erosion processes.

## Figures and Tables

**Table 1:** Particle size distribution of the experimental soil.

Soil Texture	Clay	Silt	Sand
Particle size (mm)	<0.002	0.002~0.05	0.05~0.25
Percentage (%)	8.70	54.72	36.58

**Table 2:** The unit flow discharge, slope, and sediment load of experimental design.

Unit Flow Discharge (m^2^ s^−1^)	Slope (°)	Sediment Load (kg m^−1^ s^−1^)
0.00111 (400 L/h)	6	0% *T_c_*
0.00156 (560 L/h)	9	10% *T_c_*
0.00200 (720 L/h)	12	25% *T_c_*
0.00244 (880 L/h)	15	50% *T_c_*
0.00289 (1040 L/h)	18	75% *T_c_*
0.00333 (1200 L/h)	21	90% *T_c_*
0.00378 (1360 L/h)		100% *T_c_*

**Table 3:** Statistics between measured and predicted data using the soil detachment equation in WEPP under different slope conditions.

Slope	*RE*	*MRE*	*MARE*	*R* ^2^	*NSE*	*n*
Gentle slope	−119.77 to 100	−10.36	35.57	0.9516	0.9484	147
Steep slope	−47.16 to 100	−19.15	29.24	0.9681	0.9572	147
All slope	−119.77 to 100	−14.75	32.41	0.9667	0.9611	294

Note: *RE* is relative error (%), *MRE* is mean relative error (%), *MARE* is mean absolute relative error (%), *R*^2^ is determination coefficient *NSE* is Nash-Sutcliffe efficiency, *n* is the number of data.

**Table 4:** Statistics between measured and predicted data using the soil detachment equation in EUROSEM under different slope conditions.

Slope	*RE*	*MRE*	*MARE*	*R* ^2^	*NSE*	*n*
Gentle slope	99.5237 to100	99.9	99.9	0.7772	−1.1312	147
Steep slope	99.7836 to 100	99.93	99.93	0.7874	−1.5918	147
All slope	99.5237 to 100	99.91	99.91	0.8117	−1.1402	294

**Table 5:** The correlation equations between detachment rate and sediment load for the 42 combinations of slopes and flow discharges (*D_r_* = *a − bq_s_*). The units for *D_r_* and *q_s_* are m^3^ s^−1^ m^−1^ and m^3^ m^−3^, respectively.

Flow Discharge (m^2^ s^−1^)	Bed Slope (%)	Correlation Equation	*R* ^2^	Intercept *βωv_s_T_c_* *a*	Slope *βωv_s_* **b**	Measured *βωv_s_T_c_*	Measured *βωv_s_*
0.00111	10.51	*D_r_* = 0.0010–0.0116 *q_s_*	0.9067	0.0010	0.0116	2.49 × 10^−6^	2.76 × 10^−5^
0.00111	15.84	*D_r_* = 0.0016–0.0112 *q_s_*	0.9836	0.0016	0.0112	3.65 × 10^−6^	2.77 × 10^−5^
0.00111	21.26	*D_r_* = 0.0031–0.0149 *q_s_*	0.9334	0.0031	0.0149	5.66 × 10^−6^	2.77 × 10^−5^
0.00111	26.79	*D_r_* = 0.0042–0.0159 *q_s_*	0.9549	0.0042	0.0159	7.01 × 10^−6^	2.77 × 10^−5^
0.00111	32.49	*D_r_* = 0.0051–0.0166 *q_s_*	0.9695	0.0051	0.0166	8.20 × 10^−6^	2.74 × 10^−5^
0.00111	38.39	*D_r_* = 0.0006–0.0161 *q_s_*	0.9525	0.0060	0.0161	9.94 × 10^−6^	2.74 × 10^−5^
0.00156	10.51	*D_r_* = 0.0015–0.0162 *q_s_*	0.8477	0.0015	0.0162	2.39 × 10^−6^	2.60 × 10^−5^
0.00156	15.84	*D_r_* = 0.0024–0.0142 *q_s_*	0.9505	0.0024	0.0142	3.94 × 10^−6^	2.60 × 10^−5^
0.00156	21.26	*D_r_* = 0.0041–0.0186 *q_s_*	0.9229	0.0041	0.0186	5.19 × 10^−6^	2.60 × 10^−5^
0.00156	26.79	*D_r_* = 0.0054–0.0208 *q_s_*	0.9596	0.0054	0.0208	6.24 × 10^−6^	2.67 × 10^−5^
0.00156	32.49	*D_r_* = 0.0065–0.0191 *q_s_*	0.9695	0.0065	0.0191	8.25 × 10^−6^	2.67 × 10^−5^
0.00156	38.39	*D_r_* = 0.0080–0.0222 *q_s_*	0.9781	0.0080	0.0222	9.08 × 10^−6^	2.67 × 10^−5^
0.00200	10.51	*D_r_* = 0.0023–0.0203 *q_s_*	0.8479	0.0023	0.0203	3.06 × 10^−6^	2.67 × 10^−5^
0.00200	15.84	*D_r_* = 0.0034–0.0198 *q_s_*	0.9403	0.0034	0.0198	4.14 × 10^−6^	2.70 × 10^−5^
0.00200	21.26	*D_r_* = 0.0054–0.0256 *q_s_*	0.9569	0.0054	0.0256	5.33 × 10^−6^	2.70 × 10^−5^
0.00200	26.79	*D_r_* = 0.0073–0.0275 *q_s_*	0.9897	0.0073	0.0275	6.43 × 10^−6^	2.70 × 10^−5^
0.00200	32.49	*D_r_* = 0.0080–0.0233 *q_s_*	0.9819	0.0081	0.0233	8.68 × 10^−6^	2.79 × 10^−5^
0.00200	38.39	*D_r_* = 0.0098–0.0248 *q_s_*	0.9925	0.0098	0.0248	9.63 × 10^−6^	2.72 × 10^−5^
0.00244	10.51	*D_r_* = 0.0033–0.0284 *q_s_*	0.9299	0.0033	0.0284	3.16 × 10^−6^	2.75 × 10^−5^
0.00244	15.84	*D_r_* = 0.0047–0.0263 *q_s_*	0.9894	0.0047	0.0263	4.32 × 10^−6^	2.75 × 10^−5^
0.00244	21.26	*D_r_* = 0.0063–0.0260 *q_s_*	0.9849	0.0063	0.0260	5.81 × 10^−6^	2.76 × 10^−5^
0.00244	26.79	*D_r_* = 0.0080–0.0262 *q_s_*	0.9954	0.0080	0.0262	7.25 × 10^−6^	2.76 × 10^−5^
0.00244	32.49	*D_r_* = 0.0092–0.0235 *q_s_*	0.9934	0.0092	0.0235	9.23 × 10^−6^	2.76 × 10^−5^
0.00244	38.39	*D_r_* = 0.0123–0.0278 *q_s_*	0.9893	0.0123	0.0278	1.05 × 10^−5^	2.76 × 10^−5^
0.00289	10.51	*D_r_* = 0.0050–0.0350 *q_s_*	0.9456	0.0050	0.0350	3.99 × 10^−6^	2.75 × 10^−5^
0.00289	15.84	*D_r_* = 0.0058–0.0259 *q_s_*	0.9631	0.0058	0.0259	5.60 × 10^−6^	2.75 × 10^−5^
0.00289	21.26	*D_r_* = 0.0080–0.0299 *q_s_*	0.9890	0.0080	0.0299	6.60 × 10^−6^	2.75 × 10^−5^
0.00289	26.79	*D_r_* = 0.0099–0.0301 *q_s_*	0.9763	0.0099	0.0301	7.95 × 10^−6^	2.72 × 10^−5^
0.00289	32.49	*D_r_* = 0.0124 –0.0304 *q_s_*	0.9769	0.0124	0.0304	1.05 × 10^−5^	2.81 × 10^−5^
0.00289	38.39	*D_r_* = 0.0151–0.0343 *q_s_*	0.9790	0.0151	0.0343	1.18 × 10^−5^	2.81 × 10^−5^
0.00333	10.51	*D_r_* = 0.0058–0.0411 *q_s_*	0.9590	0.0058	0.0411	3.89 × 10^−6^	2.83 × 10^−5^
0.00333	15.84	*D_r_* = 0.0072–0.0314 *q_s_*	0.9864	0.0072	0.0314	5.61 × 10^−6^	2.74 × 10^−5^
0.00333	21.26	*D_r_* = 0.0104 –0.037 *q_s_*	0.9859	0.0104	0.0370	7.10 × 10^−6^	2.84 × 10^−5^
0.00333	26.79	*D_r_* = 0.0116–0.0355 *q_s_*	0.9950	0.0116	0.0355	8.46 × 10^−6^	2.84 × 10^−5^
0.00333	32.49	*D_r_* = 0.0148–0.0373 *q_s_*	0.9849	0.0148	0.0373	1.05 × 10^−5^	2.83 × 10^−5^
0.00333	38.39	*D_r_* = 0.0173–0.0376 *q_s_*	0.9834	0.0173	0.0376	1.20 × 10^−5^	2.82 × 10^−5^
0.00378	10.51	*D_r_* = 0.0070–0.0450 *q_s_*	0.9135	0.0070	0.0450	4.29 × 10^−6^	2.72 × 10^−5^
0.00378	15.84	*D_r_* = 0.0092–0.0392 *q_s_*	0.9980	0.0092	0.0392	6.16 × 10^−6^	2.81 × 10^−5^
0.00378	21.26	*D_r_* = 0.0119–0.0427 *q_s_*	0.9939	0.0119	0.0427	7.05 × 10^−6^	2.74 × 10^−5^
0.00378	26.79	*D_r_* = 0.0147–0.0454 *q_s_*	0.9742	0.0147	0.0454	8.48 × 10^−6^	2.75 × 10^−5^
0.00378	32.49	*D_r_* = 0.0161–0.0411 *q_s_*	0.9740	0.0161	0.0411	1.00 × 10^−5^	2.77 × 10^−5^
0.00378	38.39	*D_r_* = 0.0197–0.0445 *q_s_*	0.9778	0.0197	0.0445	1.15 × 10^−5^	2.80 × 10^−5^

Where *D_r_* is soil detachment rate by rill flow, m^3^ s^−1^ m^−1^; *q_s_* is sediment load, m^3^ m^−3^.

**Table 6:** Statistics between measured and predicted data using the revised detachment equation in EUROSEM without *v_s_* under different slope conditions.

Slope	*RE*	*MRE*	*MARE*	*R* ^2^	*NSE*	*n*
Gentle slope	−477.79 to100	−17.96	65.86	0.7629	0.7602	147
Steep slope	−164.53 to 100	8.91	44.86	0.7736	0.769	147
All slope	99.52 to 100	−4.53	55.36	0.7994	0.7984	294

## Data Availability

The data presented in this study are available on request from the corresponding author.
